# *NetMHCIIpan-2.0 *- Improved pan-specific HLA-DR predictions using a novel concurrent alignment and weight optimization training procedure

**DOI:** 10.1186/1745-7580-6-9

**Published:** 2010-11-13

**Authors:** Morten Nielsen, Sune Justesen, Ole Lund, Claus Lundegaard, Søren Buus

**Affiliations:** 1 Center A for Biological Sequence Analysis, BioCentrum-DTU, Building 208, Technical University of Denmark, DK-2800 Lyngby, Denmark; 2 Laboratory of Experimental Immunology, Faculty of Health Sciences, University of Copenhagen, Denmark

## Abstract

**Background:**

Binding of peptides to Major Histocompatibility class II (MHC-II) molecules play a central role in governing responses of the adaptive immune system. MHC-II molecules sample peptides from the extracellular space allowing the immune system to detect the presence of foreign microbes from this compartment. Predicting which peptides bind to an MHC-II molecule is therefore of pivotal importance for understanding the immune response and its effect on host-pathogen interactions. The experimental cost associated with characterizing the binding motif of an MHC-II molecule is significant and large efforts have therefore been placed in developing accurate computer methods capable of predicting this binding event. Prediction of peptide binding to MHC-II is complicated by the open binding cleft of the MHC-II molecule, allowing binding of peptides extending out of the binding groove. Moreover, the genes encoding the MHC molecules are immensely diverse leading to a large set of different MHC molecules each potentially binding a unique set of peptides. Characterizing each MHC-II molecule using peptide-screening binding assays is hence not a viable option.

**Results:**

Here, we present an MHC-II binding prediction algorithm aiming at dealing with these challenges. The method is a pan-specific version of the earlier published allele-specific *NN-align *algorithm and does not require any pre-alignment of the input data. This allows the method to benefit also from information from alleles covered by limited binding data. The method is evaluated on a large and diverse set of benchmark data, and is shown to significantly out-perform state-of-the-art MHC-II prediction methods. In particular, the method is found to boost the performance for alleles characterized by limited binding data where conventional allele-specific methods tend to achieve poor prediction accuracy.

**Conclusions:**

The method thus shows great potential for efficient boosting the accuracy of MHC-II binding prediction, as accurate predictions can be obtained for novel alleles at highly reduced experimental costs. Pan-specific binding predictions can be obtained for all alleles with know protein sequence and the method can benefit by including data in the training from alleles even where only few binders are known. The method and benchmark data are available at http://www.cbs.dtu.dk/services/NetMHCIIpan-2.0

## Background

Binding of peptides to MHC II molecules play a major role in governing adaptive immune responses. They allow peptides derived from pathogens in the extracellular compartment to be presented by professional antigen presenting cells (APCs) to T helper cells of the immune system.

These T cells might in turn activate the presenting cell to kill intracellular bacterial infections. Help is also for most antigens needed to activate B cells to produce antibodies that may neutralize the pathogen. Over the last decade a number of different methods for prediction of binding to MHC II molecules have been developed, the most known being the *TEPITOPE *method [1]. Prediction of binding of peptides to MHC II is complicated by the immense polymorphism of the MHC class II alleles since the many different encoded MHC class II molecules (more than 690 different known HLA-DR alleles are known) bind very different sets of peptides. The *TEPITOPE *method covers 50 of these HLA-DR alleles. During the last decays several data driven so-called allele-specific methods have been developed for alleles where sufficient numbers of binding peptides are known. These methods cover a very broad range of different bioinformatics training algorithms including Gibbs sampler [2,3], artificial neural networks [4,5], support vector machines [6-8], hidden Markov models [9], as well as other (often exotic) motif search algorithms [10-18]. For a detailed review please refer to Nielsen et al. [19].

These methods can interpolate between peptide binding data and create predictions for peptides not present in the training set. Recently, pan-specific methods that in principle can make predictions for all alleles with known amino acid sequence have been developed [20-26]. These methods work by including information about the amino acid sequence of the MHC molecule as input to the method allowing the methods to integrate information across multiple alleles simultaneously thus boosting the predictive performance and potentially extrapolate the predictions to previously un-characterized MHC molecules. Several benchmark calculations have demonstrated the power of such pan-specific methods [27] and have shown how accurate predictions can be obtained also for alleles for which no or very limited binding data have been identified [21,28].

One of the best performing pan-specific MHC class II prediction method is the *NetMHCIIpan *method [29]. An important limiting factor for this method lies in the need for a pre-alignment of the input training data identifying the peptide-binding core prior to the training of the method. Such pre-alignments require sufficient data being available for all MHC molecules included in the training data in order to derive accurate allele-specific predictions. It has earlier been shown that this number of peptide binding data for MHC class II is of the order of many hundred [3,19], which makes it very costly to develop accurate MHC class II predictions. In order to circumvent this, we here propose a less demanding, yet highly efficient method to generate MHC class II predictors. This method is a pan-specific version of the earlier published allele-specific *NN-align *algorithm [5] and does not require any pre-alignment of the input data. The method hence has the potential to benefit also from information from alleles covered by limited binding data. Here, we demonstrate its predictive power in a series of large-scale benchmark calculations.

## Materials and methods

### Data

Quantitative peptide binding data covering 24 HLA-DR molecules were obtained from the IEDB database and combined with data from an in-house database containing MHC class II peptide binding affinity data obtained from a high-throughput peptide-binding screening assay described earlier [30]. The peptide coverage in the data set varied from a maximal coverage of 7685 peptide binding measurements for the DRB1*0101 allele to a minimal coverage of only 30 peptide binding measurements for the DRB1*1404 allele (see table 1). The peptide data were split into 5 groups used for cross validation using the approach described by Nielsen et al. [3] minimizing the sequence overlap between the training and test data. Each data set and the corresponding partitions are available online at http://www.cbs.dtu.dk/suppl/immunology/NetMHCIIpan-2.0.

**Table 1:** Quantitative HLA-DR peptide binding data

Allele	#	# bind	Allele	#	# bind
DRB1*0101	7685	4382	DRB1*1101	1794	778
DRB1*0301	2505	649	DRB1*1201	117	81
DRB1*0302	148	44	DRB1*1202	117	79
DRB1*0401	3116	1039	DRB1*1302	1580	493
DRB1*0404	577	336	DRB1*1402	118	78
DRB1*0405	1582	627	DRB1*1404	30	16
DRB1*0701	1745	849	DRB1*1412	116	63
DRB1*0802	1520	431	DRB1*1501	1769	709
DRB1*0806	118	91	DRB3*0101	1501	281
DRB1*0813	1370	455	DRB3*0301	160	70
DRB1*0819	116	54	DRB4*0101	1521	485
DRB1*0901	1520	622	DRB5*0101	3106	1280

Total				33931	13992

# is the number of peptide binding data for each allele, and #bind is the number of peptides with binding affinity stronger than 500 nM.

A large set of MHC class II ligands from the SYFPEITHI database [15] (November 2009) was used as external evaluation set. Only ligands with at least four digit HLA-DR resolution were used. All ligands included in the training data were excluded from the evaluation set. The SYFPEITHI evaluation data sets consist of 1164 MHC class II ligands, restricted to a total of 28 HLA-DR alleles (see table 2).

**Table 2:** MHC class II ligands from the SYFPEITHI database

Allele	#	Allele	#
HLA-DRB1*0101	53	HLA-DRB1*1101	35
HLA-DRB1*0102	5	HLA-DRB1*1104	8
HLA-DRB1*0301	88	HLA-DRB1*1201	11
HLA-DRB1*0401	468	HLA-DRB1*1301	16
HLA-DRB1*0402	36	HLA-DRB1*1302	19
HLA-DRB1*0403	1	HLA-DRB1*1401	9
HLA-DRB1*0404	42	HLA-DRB1*1501	22
HLA-DRB1*0405	36	HLA-DRB1*1502	3
HLA-DRB1*0701	47	HLA-DRB1*1601	2
HLA-DRB1*0801	39	HLA-DRB3*0101	2
HLA-DRB1*0802	1	HLA-DRB3*0301	5
HLA-DRB1*0803	1	HLA-DRB4*0101	6
HLA-DRB1*0901	6	HLA-DRB4*0103	2
HLA-DRB1*1001	183	HLA-DRB5*0101	18

Total			1164

A second evaluation set consisted of HLA-DR class II restricted T cell epitopes downloaded from the IEDB database June 28^th^, 2010 [31]. Also here, only epitopes with four digit HLA-DR resolution were used. As above, all epitopes included in the quantitative training data were excluded. Further, epitopes shorter than 9 or longer than 24 amino acids were excluded, since shorter peptides do not fit the 9 amino acid core of the HLA-DR binding motif, and longer peptide most likely are not experimentally characterized as minimal epitopes. This leaves us with a set of 1325 epitopes covering 42 HLA-DR alleles (see table 3).

**Table 3:** HLA-DR restriction T cell epitope from the IEDB database

Allele	#	Allele	#
HLA-DRB1*0101	125	HLA-DRB1*1103	3
HLA-DRB1*0102	4	HLA-DRB1*1104	6
HLA-DRB1*0103	5	HLA-DRB1*1201	3
HLA-DRB1*0301	173	HLA-DRB1*1301	15
HLA-DRB1*0401	342	HLA-DRB1*1302	10
HLA-DRB1*0402	33	HLA-DRB1*1303	3
HLA-DRB1*0403	14	HLA-DRB1*1401	16
HLA-DRB1*0404	46	HLA-DRB1*1404	1
HLA-DRB1*0405	21	HLA-DRB1*1405	2
HLA-DRB1*0406	6	HLA-DRB1*1501	193
HLA-DRB1*0407	4	HLA-DRB1*1502	20
HLA-DRB1*0408	2	HLA-DRB1*1503	2
HLA-DRB1*0701	56	HLA-DRB1*1601	5
HLA-DRB1*0703	1	HLA-DRB1*1602	3
HLA-DRB1*0801	4	HLA-DRB3*0101	12
HLA-DRB1*0802	2	HLA-DRB3*0202	10
HLA-DRB1*0803	2	HLA-DRB3*0301	1
HLA-DRB1*0901	13	HLA-DRB4*0101	17
HLA-DRB1*1001	4	HLA-DRB4*0103	1
HLA-DRB1*1101	88	HLA-DRB5*0101	55
HLA-DRB1*1102	1	HLA-DRB5*0102	1

Total			1325

### Method

The pan-specific *NetMHCIIpan-2.0 *method is a hybrid of the earlier published methods for pan-specific for MHC class I and class II binding, *NetMHCpan *[20,21], and *NetMHCIIpan *[29], and the *NN-align *method recently published for allele-specific MHC class II binding predictions [5]. The overall method architecture is similar to the *NN-align *method, and the manner in which the MHC polymorphism method is incorporated is similar to that of the *NetMHCpan *and *NetMHCIIpan *methods.

The method was implemented as a conventional feed-forward artificial neural network. Like the *NN-align *method, the method consists of a two-step procedure that simultaneously estimates the optimal peptide binding register (core) and network weight configuration. Initially, all network weights were assigned random values. Given this set of network weights, the core of a given peptide was identified as the highest scoring of all nonamers contained within the peptide. The score of a nonamer peptide was calculated using the conventional feed-forward algorithm. The network weights were updated using gradient descent back-propagation. Given a peptide core alignment, the weights were updated to lower the sum of squared errors between the predicted binding score and the measured binding affinity target value. A peptide core was presented to the network as described for the *NN-align *method including encoding of peptide flanking regions (PFR), PFR length and the peptide length. The MHC environment defining the peptide binding strength was implemented in terms of the MHC pseudo sequence constructed from 21 polymorphic amino acid positions in potential contact with the bound peptide as described by Nielsen et al. [29]. Two types of sequence encodings (sparse and blosum) were applied for the peptide-core and MHC pseudo sequences as described by Nielsen et al. [32]. For each peptide core, the input to the neural network thus consisted of the peptide core and MHC environment residues ((9+21) × 20 = 600 inputs), the PFRs (2 × 20 = 40 inputs), the peptide length (2 inputs), the length of the C and N terminal PFR's (2 × 2 = 4 inputs) resulting in a total of 646 input values. The peptide binding affinity IC50 values were encoded to the neural network as log-transformed values, using the relation 1-log (aff)/log(15,000), where aff is the measured binding affinity (IC50) in nM units [32]. Note, that we here use 15,000 as the base for the logarithmic transformation. This is in contrast to the 50,000 used in previous works by our group, and is chosen due to a lower sensitivity for weak binding peptides of the high-throughput peptide-binding screening assay described by Justesen et al. [30].

The networks were trained using 5-fold cross-validation. Network ensembles were trained with 40 hidden neurons. The procedure of i) identifying the optimal peptide core, and ii) updating the network weights to lower the predictive error was repeated for 500 cycles. Since the "search landscape" has a large set of local minima each with close to identical performance values, the network training was repeated 10 times, each with different initial configuration values. This led to significantly improved prediction accuracy (data not shown). In total 20 (2 encoding schemes*10 seeds) networks were created for each training/test set configuration. The binding core of a given peptide was assigned by a majority vote of the networks in the ensemble.

### Leave-one-out (LOO) network training and benchmark

Leave-one-(allele)-out experiments were conducted to investigate the predictive performance of the method in situations where binding data for a given allele was excluded from the training. Two types of LOO experiments were conducted. In the first type, peptide-binding data for a given allele were excluded from the training of the prediction method, and upon training, the predictive performance was evaluated using the peptide binding affinity values for the HLA-DR molecule in question. This is the LOO approach applied in the *NetMHCIIpan-1.0 *method [29] and allows for a direct comparison of this method to the method proposed here when trained on similar data sets.

Since many of the peptides in the training data have been measured for binding affinities to multiple alleles, the above LOO experiment can lead to a significant overestimation of the performance for a given prediction method. To reduce this bias, a second type of LOO experiment was conducted where all data representing a given peptide was excluded from the training of the prediction method. That is, if a given peptide was measured against multiple alleles including the allele in question, all these measurements were excluded from the LOO training. To avoid reducing the size of the training data too much, this second type of LOO training was performed as a three-fold cross-validation for alleles characterized by more than 200 data points.

### Performance measures

The predictive performance was measured in terms of the area under the ROC curve (AUC) value and Pearson's correlation coefficient (PCC). The receiver operating characteristic (ROC) curve is a graphical plot of the sensitivity versus the false positive rate (1 - specificity) as the discrimination threshold is varied. Through out this work, a binding threshold value of 500 nM was used to classify the peptides. The area under the ROC curve (AUC) gives an indication of the accuracy of a prediction method. An AUC value of 1 corresponds to perfect predictions and a value of 0.5 reflects random predictions. Likewise, PCC is a measure of the accuracy of a prediction method. It is obtained by dividing the covariance of the two variables by the product of their standard deviations. For perfect predictions PCC is 1 (or -1), and for random predictions PCC is 0.

### Nearest neighbor distance calculation

The distance between two MHC alleles was estimated as described by Nielsen et al. [29] using the relation $d=1-\frac{s(A,B)}{\sqrt{s(A,A)\cdot s(B,B)}}$, where s(A, B) is the BLOSUM50 similarity score between the pseudo sequences of allele A and B, respectively. Next, the nearest neighbor distance for an allele is defined as the minimal distance to any allele included in the training data set.

## Results

### Pan-specific versus allele-specific predictions

In contrast to allele-specific MHC class II prediction methods, the pan-specific method outlined here is proposed to benefit from information even from alleles covered by limited binding data. To demonstrate this, we in table 4 show the performance values obtained by the new *NetMHCIIpan-2.0 *and older *NN-align *method using 5 fold cross-validation. The NN-align method was trained in an allele-specific manner as described in by Nielsen et al. [5]. As a reference, the performance of the *TEPITOPE *method is also included in the benchmark study. The predictive performance for each HLA allele was measured in terms of the area under the ROC curve (AUC) value and Pearson's correlation coefficient (PCC).

**Table 4:** Five-fold cross-validation performance of the pan-specific *NetMHCIIpan-2.0 *method compared to the allele-specific *NN-align *and *TEPITOPE *methods on the quantitative benchmark data set

			*NN-align*		*NetMHCIIpan-2.0*		*TEPITOPE*
**Allele**	**#**	**# bind**	**PCC**	**AUC**	**PCC**	**AUC**	**AUC**

DRB1*0101	7685	4382	0.675	0.825	**0.711**	**0.846**	0.727
DRB1*0301	2505	649	0.690	0.855	**0.709**	**0.864**	0.718
DRB1*0302	148	44	0.272	0.659	**0.525**	**0.757**	
DRB1*0401	3116	1039	0.643	0.833	**0.670**	**0.848**	0.762
DRB1*0404	577	336	0.565	0.766	**0.630**	**0.818**	0.747
DRB1*0405	1582	627	**0.710**	**0.869**	0.698	0.858	0.780
DRB1*0701	1745	849	0.718	0.855	**0.740**	**0.864**	0.777
DRB1*0802	1520	431	0.518	0.778	**0.526**	**0.780**	0.645
DRB1*0806	118	91	0.744	0.902	**0.796**	**0.924**	0.884
DRB1*0813	1370	455	0.729	0.878	**0.746**	**0.885**	0.750
DRB1*0819	116	54	0.370	0.706	**0.608**	**0.808**	
DRB1*0901	1520	622	0.597	0.810	**0.634**	**0.818**	
DRB1*1101	1794	778	0.756	0.873	**0.777**	**0.883**	0.793
DRB1*1201	117	81	0.699	0.860	**0.764**	**0.892**	
DRB1*1202	117	79	0.695	0.866	**0.769**	**0.900**	
DRB1*1302	1580	493	**0.630**	**0.819**	0.634	0.825	0.596
DRB1*1402	118	78	0.623	0.825	**0.694**	**0.860**	
DRB1*1404	30	16	0.466	0.661	**0.613**	**0.737**	
DRB1*1412	116	63	0.680	0.857	**0.757**	**0.894**	
DRB1*1501	1769	709	0.641	0.815	**0.653**	**0.819**	0.731
DRB3*0101	1501	281	0.673	0.843	**0.690**	**0.850**	
DRB3*0301	160	70	0.604	0.826	**0.736**	**0.853**	
DRB4*0101	1521	485	**0.701**	**0.854**	0.675	0.837	
DRB5*0101	3106	1280	0.735	0.865	**0.765**	**0.882**	0.760

Ave	33931	13992	0.631	0.821	0.688	0.846	
Ave*			0.673	0.841	0.697	0.854	0.744
Ave**			0.580	0.797	0.679	0.837	

# gives the number of peptide binding data for each allele, #bind gives the number of peptides with a binding affinity stronger than 500 nM. *NN-align *is the method described by Nielsen et al. [5], *NetMHCIIpan-2.0 *is the method described here, and *TEPITOPE *is the method described by Sturniolo et al. [1]. Ave gives the per allele average, Ave* gives the per allele average of the 13 alleles characterized by the *TEPITOPE *method, and Ave** gives the per allele average of the 11 allele not characterized by the *TEPITOPE *method. In bold is highlighted the best performing method for each of the 24 alleles. AUC values were calculated using a binding threshold of 500 nM. Only AUC values are included for the *TEPITOPE *method since prediction values for this method are not linearly related to the binding affinity.

From the results in table 4, it is apparent that the *NetMHCIIpan-2.0 *method significantly outperforms both the *NN-align *and *TEPITOPE *methods (p < 0.01, binomial test). For 9 of the 9 alleles covered by less than 400 peptide-binding measurements, we find that *NetMHCIIpan-2.0 *outperforms *NN-align*. These results strongly indicate that *NetMHCIIpan-2.0 *is capable of benefiting from information from the multiple alleles included in the benchmark to boost the predictive performance and deliver accurate predictions also for alleles covered by limited binding data. Only for 3 out of the 24 alleles does the *NN-align *perform better than *NetMHCIIpan-2.0*. These alleles are all covered by more than 1500 peptide-binding measurements. This hence confirms the results obtained earlier for MHC class I binding predictions namely that pan-specific predictions are particularly beneficial when binding data are scarce or absent [28]. What is also clear from the data in table 4 is that the *NetMHCIIpan-2.0 *method is capable of maintaining its high performance also for alleles not characterized by the *TEPITIOPE *method.

### NetMHCpanII-1.0 versus NetMHCpanII-2.0

In the pan-specific training algorithm implemented in the *NetMHCIIpan-2.0 *method, alignment and binding affinity prediction is performed simultaneously. To further demonstrate that this approach does indeed outperform the *NetMHCIIpan-1.0 *method where the two steps were decoupled, we performed a series of LOO experiments as described in Materials and methods. In these experiments, peptide-binding data for a given allele were excluded from the training of the prediction method, and upon training, the predictive performance was evaluated using the peptide binding affinity values for the HLA-DR molecule in question. This experiment thus simulated prediction of binding to hitherto un-characterized HLA-DR molecules. The first LOO experiment was conducted based on the binding data using in the original *NetMHCIIpan *paper covering 14 HLA-DR alleles [29]. Details of this analysis are shown in table 5. The average PCC and AUC values for the 14 LOO experiments were 0.541, 0.768 and 0.606, 0.799 for the *NetMHCIIpan-1.0 *and *NetMHCIIpan-2.0 *methods, respectively. This difference is statistically highly significant (p < 0.01, binomial test). Only for one allele (DRB1*1302) did the *NetMHCIIpan-1.0 *method achieve a higher performance than *NetMHCIIpan-2.0*. These results thus demonstrate, that the training algorithm implemented in the *NetMHCIIpan-2.0 *method leads to significantly improved prediction accuracy compared to the algorithm employed in the *NetMHCIIpan-1.0 *method.

**Table 5:** LOO benchmark comparison of the pan-specific *NetMHCIIpan-2.0 *and the *NetMHCIIpan-1.0 *methods

			*NetMHCIIpan-1.0*		*NetMHCIIpan-2.0*		*TEPITOPE*
**Allele**	**#**	**#bind**	**PCC**	**AUC**	**PCC**	**AUC**	**AUC**

DRB1*0101	5166	3510	0.571	0.778	**0.627**	**0.794**	0.720
DRB1*0301	1020	277	0.465	0.746	**0.560**	**0.792**	0.664
DRB1*0401	1024	510	0.591	0.775	**0.652**	**0.802**	0.716
DRB1*0404	663	386	0.693	0.852	**0.731**	**0.869**	0.770
DRB1*0405	630	425	0.594	0.808	**0.626**	**0.823**	0.759
DRB1*0701	853	498	0.655	0.825	**0.753**	**0.886**	0.761
DRB1*0802	420	148	0.637	0.841	**0.700**	**0.869**	0.766
DRB1*0901	530	254	0.406	0.653	**0.474**	**0.684**	
DRB1*1101	950	429	0.580	0.799	**0.721**	**0.875**	0.721
DRB1*1302	498	199	0.323	**0.658**	**0.337**	0.648	0.652
DRB1*1501	934	450	0.533	0.738	**0.598**	**0.769**	0.686
DRB3*0101	549	75	0.449	0.716	**0.474**	**0.733**	
DRB4*0101	446	200	0.448	0.724	**0.515**	**0.762**	
DRB5*0101	924	478	0.627	0.831	**0.722**	**0.879**	0.686

Ave			0.541	0.768	0.606	0.799	
Ave*			0.570	0.786	0.639	0.819	0.718

The two methods are compared in a leave-one-out experiment on the peptide binding data described in the original *NetMHCIIpan *publication [29].

# is the number of peptide binding data for each allele, #bind is the number of peptides with a binding affinity stronger than 500 nM. *NetMHCIIpan-1.0 *is the method by Nielsen et al. [29], *NetMHCIIpan-2.0 *is the method described here, and *TEPITOPE *is the method by Sturniolo et al. [1]. Prediction values for *NetMHCIIpan-1.0 *were taken from [29]. Ave gives the per allele average, Ave* gives the per allele average of the 11 alleles characterized by the *TEPITOPE *method. In bold is highlighted the best performing method for each of the 14 alleles. AUC values were calculated using a binding threshold of 500 nM. Only AUC values are included for the *TEPITOPE *method since prediction values for this method are not linearly related to the binding affinity.

Next, we investigated to what extent expanding the peptide data set with broader allelic coverage and more binding data would lead to an improved predictive performance. To do this, we conducted a second series of LOO experiments comparing the LOO predictive performance of the *NetMHCIIpan-2.0 *method when trained on the old data set covering 14 HLA-DR allele (termed OLD) to its performance when trained on the extended data set covering 24 HLA-DR alleles (termed NEW). The peptide overlap in both datasets is high, and many peptides have been measured for binding affinities against multiple alleles. This peptide overlap can impose a strongly bias in the benchmark evaluation [20], and to lower this bias all peptides used to characterize a given allele were excluded from the training of the prediction method in the second LOO benchmark (for details see Materials and methods). Both the OLD and NEW methods were evaluated using the peptide binding data in the new peptide data set. The results of the extended LOO calculation are shown in table 6.

**Table 6:** The extended LOO benchmark

			OLD				NEW				*TEPITOPE*
**Allele**	**#**	**#bind**	**PCC**	**AUC**	**NN**	**dist**	**PCC**	**AUC**	**NN**	**dist**	**AUC**

DRB1*0101	7685	4382	0.567	0.767	DRB1*0401	0.352	**0.583**	**0.786**	DRB1*1402	0.322	0.727
DRB1*0301	2505	649	0.433	0.727	DRB3*0101	0.277	**0.499**	**0.765**	DRB1*0302	0.156	0.718
DRB1*0401	3116	1039	0.563	0.787	DRB1*0405	0.066	**0.594**	**0.804**	DRB1*0405	0.066	0.762
DRB1*0404	577	336	0.592	**0.806**	DRB1*0401	0.091	**0.595**	0.804	DRB1*0401	0.091	0.747
DRB1*0405	1582	627	**0.638**	0.826	DRB1*0401	0.066	0.633	**0.833**	DRB1*0401	0.066	
DRB1*0701	1745	849	**0.659**	**0.831**	DRB1*0901	0.504	0.648	0.826	DRB1*0901	0.504	0.780
DRB1*0802	1520	431	**0.380**	**0.710**	DRB1*1101	0.111	0.369	0.692	DRB1*0813	0.041	0.777
DRB1*0901	1520	622	**0.539**	0.757	DRB5*0101	0.431	0.517	**0.762**	DRB5*0101	0.431	0.645
DRB1*1101	1794	778	**0.602**	**0.799**	DRB1*1302	0.084	0.460	0.741	DRB1*1302	0.084	
DRB1*1302	1580	493	**0.338**	**0.691**	DRB1*1101	0.084	0.323	0.671	DRB1*1101	0.084	0.793
DRB1*1501	1769	709	**0.568**	**0.775**	DRB1*0404	0.295	0.525	0.756	DRB1*0404	0.295	0.596
DRB3*0101	1501	281	0.339	0.672	DRB1*0301	0.277	**0.374**	**0.702**	DRB3*0301	0.223	0.731
DRB4*0101	1521	485	0.506	0.753	DRB1*0404	0.397	**0.518**	**0.766**	DRB1*0404	0.397	
DRB5*0101	3106	1280	0.547	0.781	DRB1*1101	0.295	**0.608**	**0.813**	DRB1*1101	0.295	


DRB1*0302	148	44	0.396	0.729	DRB1*0301	0.156	**0.542**	0.759	DRB1*1402	0.119	**0.760**
DRB1*0806	118	91	0.670	0.886	DRB1*0802	0.107	**0.703**	**0.902**	DRB1*0802	0.107	
DRB1*0813	1370	455	**0.505**	0.735	DRB1*0802	0.041	**0.340**	0.666	DRB1*0802	0.041	**0.884**
DRB1*0819	116	54	**0.567**	0.789	DRB1*0802	0.107	**0**.566	**0.813**	DRB1*0813	0.083	0.750
DRB1*1201	117	81	**0.626**	0.786	DRB1*1101	0.445	0.609	**0.798**	DRB1*1202	0.045	
DRB1*1202	117	79	0.623	0.814	DRB1*1101	0.399	**0.713**	**0.879**	DRB1*1201	0.045	
DRB1*1402	118	78	0.570	0.793	DRB1*1101	0.148	**0.659**	**0.846**	DRB1*0302	0.119	
DRB1*1404	30	16	0.393	0.594	DRB1*0404	0.311	**0.646**	**0.679**	DRB1*0806	0.240	
DRB1*1412	116	63	0.640	0.845	DRB1*0802	0.180	**0.738**	**0.897**	DRB1*0813	0.139	
DRB3*0301	160	70	0.395	0.738	DRB3*0101	0.223	**0.545**	**0.765**	DRB3*0101	0.223	

Ave			0.527	0.766			0.554	0.780			
Ave*			0.543	0.779			0.529	0.774			0.744
Ave**			0.539	0.771			0.606	0.800			

The predictive performance of the pan-specific NN-align method when trained in a leave-one-out experiment and evaluated on the 24 alleles included in the new peptide binding data set.

# is the number of peptide binding data for each allele, #bind is the number of peptides with a binding affinity stronger than 500 nM. OLD is the method described here trained on the old peptide data set, NEW is the method described here trained on the new data set, and *TEPITOPE *is the method by Sturniolo et al. [1]. NN is the nearest neighbor as defined by the pseudo sequence distance, and dist is the nearest neighbor distance calculated as described in Materials and methods. Ave is the per allele average, Ave* is the per allele average of the 13 alleles characterized by the *TEPITOPE *method, and Ave** is the per-allele average performance of the 10 alleles included in the new peptide binding data set. In bold is highlighted the best performing method for each of the 24 alleles. AUC values were calculated using a binding threshold of 500 nM. Only AUC values are included for the *TEPITOPE *method since prediction values for this method are not linearly related to the binding affinity. The double line separates the 10 novel alleles from the original 14 alleles included in the development of the *NetMHCIIpan-1.0 *method.

The results presented in table 6 clearly demonstrate that the enrichment of novel peptide binding data with a broader allelic coverage leads to an improved predictive performance of the pan-specific prediction method. We can quantify to what degree an allele will benefit from other alleles being present in the training data by calculating its distance to the nearest neighbor in the training data (see Materials and methods). Earlier work has demonstrated that this distance measure correlates strongly with the performance of pan-specific prediction methods [20,21]. For 10 of the 11 alleles, where including the novel 10 alleles has decreased the nearest neighbor distance, the NEW method has a higher AUC predictive performance compared to the OLD. For the remaining 13 alleles, the 10 novel alleles have not altered the nearest neighbor distance and the performance of the two methods is similar. This strongly underlines the essential prerequisite for accurate pan-specific prediction methods demonstrated earlier for MHC class I, namely a population of the close neighborhood of un-characterized MHC molecules [20,28].

### Lin benchmark

The Lin benchmark consists of binding affinities of 103 overlapping peptides to seven common HLA-DR molecules (DRB1*0101, 0301, 0401, 0701, 1101, 1301, and 1501). The results of this benchmark are shown in table 7.

**Table 7:** Predictive performance in terms of the AUC on the Lin benchmark data set

Allele	*NetMHCIIpan-2.0*	*NetMHCIIpan-1.0*	*TEPITOPE*	Multipred_SVM	SVMHC
DRB1*0101	0.883	0.847	**0.919**	0.860	0.860
DRB1*0301	0.716	0.668	0.718	**0.800**	0.690
DRB1*0401	**0.846**	0.815	0.745	0.650	0.750
DRB1*0701	**0.878**	0.852	0.715	0.700	0.740
DRB1*1101	**0.884**	0.821	0.824	0.780	0.830
DRB1*1301	**0.729**	0.715	0.718	0.630	0.720
DRB1*1501	**0.838**	0.791	0.737	0.620	0.660

Ave	0.825	0.787	0.768	0.720	0.750

The AUC was calculated using the following binding affinity threshold values for each of the 7 alleles: DRB1*0101, 0401, 0701, and 1501 threshold = 100 nM, DRB1*0301, 1101, and 1301, threshold = 1000 nM. The performance values for Multipred_SVM and SVMHC were taken from Nielsen et al. [5]. *TEPITOPE *is the method described by Sturniolo et al. [1]. *NetMHCIIpan-2.0 *is the pan-specific method described here, and *NetMHCIIpan-1.0 *is the pan-specific method by Nielsen et al. [29]. For each allele, the best performing method is highlighted in bold.

The results in table 7 clearly show that *NetMHCIIpan-2.0 *outperforms both the earlier *NetMHCIIpan-1.0 *method, as well as the other methods included in the benchmark.

### Identifying MHC class II ligands and T cell epitopes

The performance of the *NetMHCIIpan-2.0 *method on the large set of SYFPEITHI ligand and IEDB T cell epitope data was next investigated. The benchmark was performed as described by Nielsen et al. [29]. The ligand source protein was split into overlapping peptides of the length of the ligand/epitope. All peptides except the annotated HLA ligand/epitope were taken as negatives. This is a very stringent assumption since suboptimal peptides sharing the ligand binding-core are counted as negatives even though they could be presented on the HLA molecule. Thus, this setup is likely to underestimate the predictive performance, but the effect should be equal for all methods compared in the benchmark. For each protein-HLA ligand/epitope pair, the predictive performance was estimated as the AUC value. Table 8 gives a summary of the performance of this benchmark calculation (details can be found in Additional file 1: Table S1).

**Table 8:** Endogenous HLA-DR ligand benchmark

SYF	#	*NetMHCIIpan-1.0*	*NetMHCIIpan-2.0*	*TEPITOPE*
Ave per ligand	1164	0.800	**0.829**	
Ave per allele	28	0.788	**0.797**	
In TEPITOPE	17	0.768	0.786	**0.799**
!In TEPITOPE	11	**0.819**	0.814	

**IEDB**	**#**	***NetMHCIIpan-1.0***	***NetMHCIIpan-2.0***	***TEPITOPE***

Ave per epitope	1325	0.729	**0.751**	
Ave per allele	42	0.759	**0.781**	
In TEPITOPE	20	0.745	0.747	**0.755**
!In TEPITOPE	22	0.772	**0.812**	

*NetMHCIIpan-1.0 *is the method described by Nielsen et al. [29], *NetMHCIIpan-2.0 *is the pan-specific method described here, and TEPITOPE is the method described by Sturniolo et al. [1]. Ave per ligand/epitope gives the average AUC over the 1164/1325 ligands/epitopes in the benchmark data set. Ave per allele gives the average over the per allele averaged AUC values. In *TEPITOPE *gives the per allele average of the subset of alleles characterized by the *TEPITOPE *method, and !In TEPITOPE give the per-allele average performance of the alleles not characterized by the *TEPITOPE *method. AUC values were calculated as described in the text. For each benchmark subset, the best performing method is highlighted in bold.

Both the SYFPEITI ligand and IEDB epitope benchmarks show that the *NetMHCIIpan-2.0 *method performs better than the original *NetMHCIIpan-1.0 *method. On the per ligand basis, the *NetMHCIIpan-2.0 *method significantly outperforms *NetMHCIIpan-1.0 *for both data sets (p < 0.01, binomial test excluding ties). In terms of the per-allele performance, the *NetMHCIIpan-2.0 *also achieved a higher performance than the *NetMHCIIpan-1.0 *method. This difference is however not statistically significant (p > 0.1 binomial test, in both cases). For the alleles characterized by the *TEPITOPE *method, the *TEPITOPE *method achieves the highest performance of the three methods for both data sets. This difference, however, is not statistically significant (p > 0.5 in all cases, binomial test). For alleles not characterized by *TEPITOPE*, the *NetMHCIIpan-2.0 *method significantly outperform *NetMHCIIpan-1.0 *for the IEDB data set (p < 0.01, binomial test), whereas the two methods for this set of alleles achieve a similar predictive performance when evaluated on the SYFPEITHI dataset.

**Figure 1:**
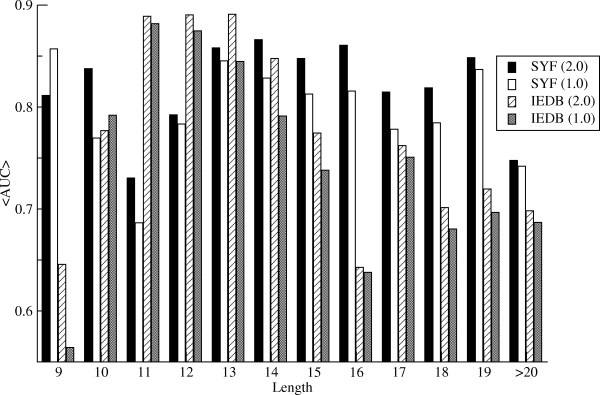
**Histogram of the predictive performance measured in terms of the AUC value for the ligands/epitopes in the SYFPEITHI/IEDB dataset as a function of the peptide length**. 2.0 refers to the pan-specific method developed here, and 1.0 refers to the *NetMHCIIpan-1.0 *method. SYF refers to the SYFPEITHI ligand data set, and IEDB refers to the IEDB T cell epitope data set.

We next investigated how the predictive performance of the *NetMHCIIpan-2.0 *method depended on the length of the ligand/epitope under investigation. Figure 1 shows a histograms of the average AUC values for the *NetMHCIIpan-2.0 *(named 2.0) and *NetMHCIIpan-1.0 *(named 1.0) methods as a function of the ligand/epitope length for the SYFPEITHI and IEDB data sets, respectively.

Figure 1 clearly demonstrates that the *NetMHCIIpan-2.0 *method, for the majority of peptide lengths, outperforms the *NetMHCIIpan-1.0 *method. Only for very short peptides (length equal to 9 for the SYFPEITHI data set and length equal to 10 for the IEDB data set) does the *NetMHCIIpan-1.0 *achieve the highest AUC value. What is also clear for the IEDB data set is that both methods achieve their highest predictive performance for peptides of length less than 15 amino acids. The average AUC for epitopes with a length less then 15 amino acids is 0.823. This values is significantly higher than the average AUC for epitopes with a length greater than 15 (0.704, p < 0.005, t-test). This difference is not observed for the SYFPEITHI ligand data set, hence strongly suggesting that the longer epitopes in the IEDB data set are not "true" epitopes in the sense of defining the minimal HLA restriction element.

## Discussion

Development of accurate prediction algorithms for MHC class II binding is complicated by the fact that the MHC class II molecule has an open binding cleft, and that peptide binders are accommodated in the binding cleft in a binding register that *a priori *is unknown. Training of methods for prediction of peptide-MHC class II binding hence rely on either a two step procedure where first the binding register is identified and next the aligned peptides are used to train the binding prediction algorithm or a procedure where these two steps are integrated and performed simultaneously.

We have earlier shown that developing allele-specific prediction methods for MHC class II binding using the latter approach leads to higher prediction accuracy [3,5]. We have further for MHC class I demonstrated that training the predictors in a pan-specific manner, incorporating all binding data across multiple MHC molecules simultaneously in the training, leads to a significant boost in the predictive performance in particular for MHC molecules characterized by few or no binding data [20-22,28].

Based on these findings, we have in this paper developed a pan-specific method for prediction of MHC class II binding affinities. The method was trained on binding data covering multiple MHC class II simultaneously, and does not require any prior alignment or binding register-identification. The method was evaluated in several large-scale benchmarks and shown consistently to outperform all other methods investigated, including state-of the-art allele-specific (*NN-align *[5]) and pan-specific (*NetMHCIIpan *[29]) methods, as well as and the well-known *TEPITOPE *method [1]. In particular, it was demonstrated that the proposed method due to its pan-specific nature could boost performance for alleles characterized by limited binding data, and in such cases significantly out-perform allele specific methods. The method thus demonstrates great potential for efficient boosting of the accuracy of MHC class II binding prediction, as accurate predictions can be achieved for novel alleles at a highly reduced experimental cost, and pan-specific binding predictions can be obtained for all alleles with known protein sequence by a method trained using data with limited allelic coverage.

When benchmarked on large data sets of know HLA-DR ligands and epitopes, the method was shown to have a predictive performance comparable to that of *TEPITOPE *for alleles covered by this method, and maybe more important maintain this high performance also for alleles not described by the *TEPITOPE *method.

For MHC class I, we have earlier demonstrated that a pan-specific predictor can benefit from being trained on cross-loci (and cross-species) peptide binding data [20]. The development of a cross-loci model for HLA class II is complicated by the fact that the HLA-DRA molecule is close to monomorphic (only two allelic version exists). This is in contrast to HLA-DP and HLA-DQ where both the α and β chains are highly polymorphic. Moreover, the structures of the HLA molecules are less conserved across the three loci for class II compared to class I, and finally very limited peptide binding data have been generated characterizing the different HLA-DP and DQ molecules. As of September 2010, only five HLA-DP and six HLA-DQ alleles have been characterized in the IEDB database with more than 200 peptide-binding measurements [31]. Nonetheless, large amounts of peptide binding data for the HLA-DP and HLA-DQ loci will most likely become available in the near future providing a broader allelic coverage, and future evaluations will demonstrate if also MHC class II binding prediction algorithms using training algorithms like the one outlined in this work, will benefit from pan-specific training across the different loci.

The method and benchmark data sets described in this work are available at http://www.cbs.dtu.dk/services/NetMHCIIpan-2.0 (method) and http://www.cbs.dtu.dk/suppl/immunology/NetMHCIIpan-2.0 (benchmark data).

## Competing interests

The authors declare that they have no competing interests.

## Authors' contributions

MN designed, trained and evaluated the NetMHCIIpan-2.0 method. MN wrote the manuscript draft. SB and SJ made MHC peptid- binding data for the 10 novel MHC molecules. All authors contributed to and approved the final manuscript.

## Supplementary Material

Additional file 1 **HLA-DR ligand and T cell epitope benchmark**. The per-allele AUC performance values of the *NetMHCIIpan-1.0*, *NetMHCIIpan-2.0*, and *TEPITOPE *methods on the HLA-DR ligands and T cell epitope benchmark data sets. Click here for file
