# Supplementary material for: NetMHCIIpan-2.0 - Improved pan-specific HLA-DR predictions using a novel concurrent alignment and weight optimization training procedure
Source: Immunome Res. 2010 Nov 13;6:9. doi: 10.1186/1745-7580-6-9 (PMC2994798; doi:10.1186/1745-7580-6-9)
Supplement: Additional file 1 — HLA-DR ligand and T cell epitope benchmark. The per-allele AUC performance values of the NetMHCIIpan-1.0, NetMHCIIpan-2.0, and TEPITOPE methods on the HLA-DR ligands and T cell epitope benchmark data sets. [file 1745-7580-6-9-S1.PDF]

Additional file Table S1. HLA-DR ligand and T cell epitope benchmark.

(A) SYFPEITHI				
	#	*NetMHCIIPan-1.0*	*NetMHCIIpan-2.0*	*TEPITOPE*
HLA-DRB1*0101	53	0.828	0.835	0.834
HLA-DRB1*0102	5	0.878	0.927	0.879
HLA-DRB1*0301	88	0.710	0.789	0.673
HLA-DRB1*0401	468	0.824	0.875	0.834
HLA-DRB1*0402	36	0.723	0.667	0.883
HLA-DRB1*0403	1	0.991	0.845	
HLA-DRB1*0404	42	0.794	0.765	0.776
HLA-DRB1*0405	36	0.850	0.856	0.808
HLA-DRB1*0701	47	0.708	0.744	0.696
HLA-DRB1*0801	39	0.656	0.643	0.697
HLA-DRB1*0802	1	0.982	0.978	0.914
HLA-DRB1*0803	1	0.659	0.292	
HLA-DRB1*0901	6	0.876	0.957	
HLA-DRB1*1001	183	0.869	0.866	
HLA-DRB1*1101	35	0.848	0.896	0.833
HLA-DRB1*1104	8	0.919	0.911	0.870
HLA-DRB1*1201	11	0.818	0.863	
HLA-DRB1*1301	16	0.683	0.724	0.824
HLA-DRB1*1302	19	0.551	0.561	0.740
HLA-DRB1*1401	9	0.717	0.810	
HLA-DRB1*1501	22	0.699	0.671	0.717
HLA-DRB1*1502	3	0.569	0.665	0.768
HLA-DRB1*1601	2	0.943	0.849	
HLA-DRB3*0101	2	0.957	0.971	
HLA-DRB3*0301	5	0.830	0.948	
HLA-DRB4*0101	6	0.555	0.726	
HLA-DRB4*0103	2	0.795	0.827	
HLA-DRB5*0101	18	0.826	0.847	0.838
Ave per ligand	1164	0.800	0.829	
Ave per allele	28	0.788	0.797	
In Tepitope	17	0.768	0.786	0.799
! In Tepitope	11	0.819	0.814	

(B) IEDB				
Allele	#	*NetMHCIIpan-1.0*	*NetMHCIIpan-2.0*	*TEPITOPE*
HLA-DRB1*0101	125	0.776	0.810	0.808
HLA-DRB1*0102	4	0.854	0.879	0.841
HLA-DRB1*0103	5	0.756	0.667	
HLA-DRB1*0301	173	0.673	0.683	0.636
HLA-DRB1*0401	342	0.757	0.775	0.750
HLA-DRB1*0402	33	0.547	0.570	0.571
HLA-DRB1*0403	14	0.888	0.896	
HLA-DRB1*0404	46	0.743	0.744	0.733
HLA-DRB1*0405	21	0.642	0.626	0.732
HLA-DRB1*0406	6	0.793	0.741	
HLA-DRB1*0407	4	0.793	0.668	
HLA-DRB1*0408	2	0.979	0.986	0.930
HLA-DRB1*0701	56	0.760	0.742	0.734
HLA-DRB1*0703	1	0.913	0.896	0.914
HLA-DRB1*0801	4	0.655	0.663	0.586
HLA-DRB1*0802	2	0.940	0.754	0.830
HLA-DRB1*0803	2	0.919	0.852	
HLA-DRB1*0901	13	0.610	0.738	
HLA-DRB1*1001	4	0.744	0.875	
HLA-DRB1*1101	88	0.775	0.815	0.757
HLA-DRB1*1102	1	0.665	0.493	0.858
HLA-DRB1*1103	3	0.681	0.510	
HLA-DRB1*1104	6	0.729	0.807	0.781
HLA-DRB1*1201	3	0.749	0.970	
HLA-DRB1*1301	15	0.622	0.632	0.768
HLA-DRB1*1302	10	0.722	0.860	0.827
HLA-DRB1*1303	3	0.719	0.604	
HLA-DRB1*1401	16	0.731	0.789	
HLA-DRB1*1404	1	0.986	0.956	
HLA-DRB1*1405	2	0.871	0.839	
HLA-DRB1*1501	193	0.715	0.722	0.689
HLA-DRB1*1502	20	0.660	0.681	0.604
HLA-DRB1*1503	2	0.708	0.874	
HLA-DRB1*1601	5	0.779	0.724	
HLA-DRB1*1602	3	0.912	0.984	
HLA-DRB3*0101	12	0.894	0.895	
HLA-DRB3*0202	10	0.424	0.539	
HLA-DRB3*0301	1	0.890	0.966	
HLA-DRB4*0101	17	0.471	0.789	
HLA-DRB4*0103	1	0.946	0.991	
HLA-DRB5*0101	55	0.770	0.802	0.746
HLA-DRB5*0102	1	0.728	0.987	
Ave per ligand	1325	0.729	0.751	
Ave per allele	42	0.759	0.781	
In Tepitope	20	0.745	0.747	0.755

! In Tepitope	22	0.772	0.812

(A) The 1164 SYFPEITHI HLA-DR ligands (B) the 1325 IEDB HLA-DR T cell epitopes. *NetMHCIIpan-1.0* is the method described by Nielsen et al. [29], *NetMHCIIpan-2.0* is the pan-specific method described here, and *TEPITOPE* is the method described by Sturniolo et al. [2]. Ave per ligand/epitope gives the average AUC over the 1164/1325 ligands/epitopes in the benchmark data set. Ave per allele gives the average over the per allele averaged AUC values. In TEPITOPE gives the per allele average of the subset of alleles characterized by the TEPITOPE method, and !In TEPITOPE give the per-allele average performance of the alleles not characterized by the TEPITOPE method. AUC values were calculated as described in the text.
